# Caring Text Messages for Suicide Prevention in Urban American Indian Youth: Protocol for a Randomized Controlled Trial

**DOI:** 10.2196/71344

**Published:** 2025-09-26

**Authors:** Erin Renee Morgan, Marija Bogic, Luciana Hebert, Erin Poole, Nichole Tsosie, Nathania Tsosie, Kandyce Garcia, Marcia O'Leary, Raeann Mettler, Gina Johnson, Linda Son-Stone, Tassy Parker, Dedra Buchwald, Spero Manson

**Affiliations:** 1 Institute for Research and Education to Advance Community Health Washington State University Everett, WA United States; 2 Department of Medical Education and Clinical Sciences Elson S. Floyd College of Medicine Washington State University Spokane, WA United States; 3 Centers for American Indian and Alaska Native Health Colorado School of Public Health University of Colorado Anschutz Medical Campus Aurora, CO United States; 4 First Nations Community HealthSource Albequerque, NM United States; 5 Center for Native American Health University of New Mexico Albuquerque, NM United States; 6 Missouri Breaks Industries Research Incorporated Rapid City, SD United States; 7 Oyate Health Center Rapid City, SD United States; 8 Neurosciences Institute Department of Neurological Surgery, School of Medicine University of Washington Seattle, WA United States

**Keywords:** suicide prevention, mental health, depression, American Indian, technology intervention

## Abstract

**Background:**

American Indian (AI) young adults in urban areas have many cultural strengths but also face unique challenges. Cultural norms within their communities strongly emphasize relationships. Previous research has found that receiving occasional positive and nondemanding messages—caring text messages—can be beneficial among people experiencing suicidality.

**Objective:**

To ameliorate increasing rates of suicide and suicidality among AI young adults, we implemented a caring text message intervention designed to increase social connectedness.

**Methods:**

This 2-arm, double-blinded, randomized controlled trial is being implemented at 2 clinical sites with large AI populations. Partnering with clinics in Albuquerque, New Mexico, and Rapid City, South Dakota, we are recruiting AI adults aged 18-34 years to participate in a caring text message study. During the baseline visit, participants complete several surveys and an interview with study staff to understand their history of suicidal behavior. After completion of the baseline visit, participants are randomized to receive the intervention—approximately 30 caring text messages—or treatment as usual. The text messaging platform selected for this study allows bidirectional messaging; while there is no expectation that participants respond, they can provide feedback or seek additional resources. Participants are followed up at 6 and 12 months postbaseline. At the final 12-month follow-up visit, they complete many of the same surveys and participate in an interview to ascertain suicidality since their initial visit. The primary outcomes of interest are suicide-related behaviors—suicidal ideation, suicide planning, suicide attempt, or thoughts and actions requiring hospitalization. Secondary outcomes include social connectedness and other measures of mental health. We will use an intention-to-treat analysis with logistic and linear regression to calculate odds ratios and risk differences (95% CIs) for binary and continuous outcomes.

**Results:**

As of June 2025, the New Mexico site has finished recruitment and follow-up assessments. The South Dakota site is still enrolling participants and has conducted the first follow-up assessments. The project has been well-received by participants.

**Conclusions:**

This randomized controlled trial will evaluate whether a caring text message intervention is effective in reducing suicidality among AI young adults in urban areas. Participants have received the culturally tailored caring text messages. This trial will help establish whether caring text messages are an effective strategy for reducing suicidal behaviors and promoting feelings of connectedness among AI young adults.

**Trial Registration:**

ClinicalTrials.gov NCT03136094; http://clinicaltrials.gov/show/NCT03136094

**International Registered Report Identifier (IRRID):**

DERR1-10.2196/71344

## Introduction

### Background

American Indian (AI) communities have a rich and diverse cultural heritage that is deeply rooted in their connection to the land, their families, and their communities [1-3]. These strengths, combined with their traditional values and beliefs, have enabled them to withstand centuries of colonization, oppression, forced assimilation, and the loss of land and resources [4]. Despite these strong cultural foundations, the rate of suicide among AI youth and young adults remains disproportionately high compared to other racial and ethnic groups in the United States [5]. It is a leading cause of mortality in many AI communities, particularly among individuals aged 10-34 years, making it a major public health concern for AI people [5]. This concern is especially pronounced among urban AI young adults, for whom risk and protective factors may differ from both non-AI peers living in urban areas and rural AI peers [6,7].

As concerns around mental health, depression, and suicidality continue to grow, the integration of behavioral health services into primary care has become increasingly common. One method of integration is through an evidence-based practice, screening, brief intervention, and referral to treatment (SBIRT) [8]. In a typical SBIRT model, patients seeking care from their primary care provider are routinely screened for substance use disorders and mental health conditions such as depression and anxiety. If they screen positive, a colocated behavioral health provider renders a brief intervention and refers the patient to further treatment [8].

The interpersonal theory of suicide is particularly relevant for young, urban AI populations, as it identifies thwarted belongingness as contributing to suicidal ideation [9,10]. Interventions that increase feelings of belonging and reduce feelings of loneliness can be effective in reducing suicide risk [11]. Previous work has found that while social support was not highly correlated with suicidality among rural AI and Alaska Native (AIAN) youth, it protects against depressive symptoms and suicidality among urban AIAN youth [6]. Community connectedness is a major cultural strength among AIAN people. Increasing people’s sense of belonging and connection to their heritage and culture may be particularly effective for improving mental health outcomes and reducing the risk of suicide [12].

Given the importance of social connections in mitigating depressive symptoms and suicidal behaviors, previous studies have sought to supplement the SBIRT protocol with caring text messages [13-15]. This text message–based intervention sends participants messages of support and encouragement periodically throughout the year [11], a modern modification of earlier interventions that delivered similar styles of message through letter or postcard [16,17]. Due to the inequitable burden of suicide, unique risk factors, and limited availability of data regarding suicide prevention—including a dearth of culturally relevant interventions—we focused our efforts on AI young adults in urban areas. Community members and our academic team cocreated brief, powerful, site-specific text messages that highlight cultural strengths or impart wisdom of local adults, and combined them with an SBIRT protocol implemented at 2 clinical sites serving AIAN people. Our study assesses the program’s effectiveness in reducing depressive symptoms, suicidal ideation, and suicidal behaviors among a group of at-risk young adults.

### Objective

This study is part of a large AIAN-specific federal research program to address youth suicide, called the Collaborative Hubs to Reduce Burden of Suicide among American Indian and Alaska Native Youth, which has several aims [18]. Our double-blinded, 2-armed randomized controlled trial is designed to test the effectiveness of a 12-month intervention that combined caring text messages with SBIRT in reducing self-reported suicide ideation, attempts, and hospitalizations, and in increasing social connectedness and retention compared to treatment as usual among urban AI young adults. This paper describes the design and implementation of our randomized controlled trial, which seeks to increase resilience through social connectedness and engagement, thereby reducing suicidal ideation, attempts, and hospitalizations. We also detail the measures and procedures of the systematic economic evaluation of the SBIRT and caring text components of the intervention to determine their relative effects on the use of health care resources and quality of life.

## Methods

### Setting and Participants (Study Sites)

Participants are recruited from 2 partner sites: First Nations Community HealthSource (FNCH) in Albuquerque, New Mexico, and Oyate Health Center (OHC) in Rapid City, South Dakota, operated by the Great Plains Tribal Leaders’ Health Board. Both health centers are funded in part by the Indian Health Service (IHS). As a federally qualified health center, FNCH serves all patients, while OHC primarily serves tribal members eligible to receive services through IHS. Both offer a variety of services, including primary care, behavioral health, women’s health, and urgent care or same-day services.

### Eligibility

The population of interest for this study is young AI adults at mild, moderate, or severe risk for suicide. To participate, an individual must self-identify as AI, be between 18 and 34 years of age, and understand written and spoken English. They must complete the SBIRT process with clinic staff and screen positive for elevated risk of suicide or depression, but not be in danger of imminent self-harm. In addition, the participant must have a cell phone that can receive text messages and be willing to be contacted via text message. Participants who have contraindications (ie, are hospitalized) or are cognitively impaired are not eligible.

#### Recruitment and Retention (Recontact Protocols)

As part of the regular appointment process at FNCH, patients are asked to fill out the FNCH Healthy Lifestyle Questionnaire (HLQ), which includes the Columbia-Suicide Severity Rating Scale and the Patient Health Questionnaire screenings for suicide risk and depression. The HLQ is completed by newly enrolled patients at the first appointment of the calendar year and every 3 months thereafter. Trained clinic staff review and score the completed HLQ during the patient’s visit, and this information is recorded in the electronic health record (EHR) and shared with the clinical provider. Depending on the score, the provider may offer treatment and/or referral for services. Study eligibility initially hinged on scoring positive for suicide risk (low, moderate, or severe) or depression. The clinic staff or provider informs the patient about the study. Alternatively, the patient could learn about the study from a flyer posted in the clinic. If interested, the patient was connected immediately to study staff through a warm hand-off. If a patient met eligibility criteria, the site coordinator proceeded with the informed consent procedures and the baseline interview. If the patient was unable to complete baseline procedures at that time due to time constraints, the site coordinator scheduled a time within a week that is convenient for the potential participant. In addition to screening patients referred to the study through SBIRT, site staff used medical records to identify potentially eligible patients. Patients who have not recently attended the clinic and have no HLQ assessment record in their EHR in the previous 3 months were contacted by study staff and screened for eligibility.

Patients—referred to as “relatives” by local staff, which underscores their shared heritage—receiving care at OHC complete routine depression screening as part of most health care encounters. Individuals who report moderate levels of depression or mild, moderate, or severe risk for suicide were referred to behavioral health. The behavioral health provider could offer treatment or referral for services. The relative was informed about the present study. If they were eligible and interested, their contact information was shared with research staff, who are not OHC employees. Study staff follow up with OHC referrals and invite relatives (patients) to participate in the study. Study staff scheduled a time to complete informed consent and the baseline interview.

While the text message intervention contacted participants in the treatment group, responses were unnecessary unless scheduled, requested, or activated by an immediate crisis. However, participants frequently respond to the scheduled messages, usually expressing gratitude or mild distress. To maintain contact and maximize retention, participants in the control group and the treatment group were contacted by the study team at 6 months postbaseline assessment to confirm their contact information. Ten months after enrollment, site staff were permitted to begin contacting participants to schedule or confirm the 12-month follow-up assessment. Participants received text messages (email or private messaging for those who can no longer use text messaging) asking them to confirm the follow-up assessment appointment. If site staff do not receive a response, they contact the alternate contact provided by all participants at the baseline assessment to try and ascertain updated contact information. If a participant does not respond to the reminders and misses their scheduled appointment, the site staff will continue to try and contact them for up to 6 months after their due date, unless they decline further contact. Eighteen months after enrollment, participants were considered lost to follow-up.

If a participant became ineligible during the study (eg, incarcerated), they were withdrawn, and all study procedures were halted. Withdrawn participants who subsequently become eligible and wish to rejoin the study were required to contact site staff and then be reconsented to be re-enrolled. If a withdrawn participant was incarcerated, the study coordinator made 3 attempts to contact them upon learning of their potential release from the participant or an emergency contact. Upon being re-enrolled, a participant restarted the study procedures. At the time that enrollment concluded, only 1 individual was lost to follow-up and subsequently requested to re-enroll in the study.

#### Assessment Procedures

After obtaining informed consent, the study staff conducted the baseline assessment interview. If the participant was unable to complete the interview on the same day, the interview was scheduled within 1 week and can be completed in person, through a secure video platform, or over the phone. The assessment consisted of a staff-administered interview and self-administered surveys and took approximately 1.5 hours to complete. Study data were collected and organized using Research Electronic Data Capture (REDCap) tools, which are hosted at Washington State University [19]. One exception was the Suicide Attempt Self-Injury (SASI), which was collected by interview, recorded on paper, reviewed, and then entered electronically after a study visit.

#### Randomization

Randomization to the intervention group or the treatment-as-usual (TAU) control group occurred after the baseline assessment had been completed. A biostatistician not involved with data collection programmed the randomization module in REDCap to enable site coordinators to access the assignment for each participant in real time. Randomization was stratified by site. To prevent bias that might arise when study coordinators intentionally or unintentionally anticipate an upcoming randomization assignment, assignments were blocked in randomly patterned groups of 2, 4, or 6, with assignments for each arm distributed evenly within each block. Follow-up interviews for the SASI were completed by staff blinded to the participants’ treatment allocation.

### Treatment Groups

#### Resources for All Participants

Study staff provided a “care card” to all participants at baseline, which detailed clinic contact information, business hours, and provided a list of local resources for mental health and traditional healing. This care card was developed in collaboration with behavioral and primary care staff at the clinics to ensure inclusion of appropriate resources. To protect patient privacy, the care cards did not mention the research study. Participants in both groups also received text messages to verify contact details at 6 months and to remind them of and confirm their 12-month follow-up interview.

#### Treatment as Usual

All participants in the study, regardless of their assigned treatment group, continued to receive the standard treatment normally provided by the clinic, including mental health services, medication management, substance abuse treatment, and case management. Data about the type and frequency of clinic visits by study participants during the study enrollment period were collected by the study team from the participants’ EHR after their study completion.

#### Caring Texts Intervention

In addition to receiving TAU, participants assigned to the caring texts intervention group also received 28-30 supportive and nondemanding text messages; examples of these messages are available in Table 1, and the complete schedule of messages is available in Multimedia Appendix 1. The number of messages sent depended on the date of enrollment, the participant’s birthday, anniversaries, holidays, and seasonal and local events. These messages were sent over 12 months, namely, daily for the first 3 days, then 6 weekly messages, 6 biweekly messages, and 6-monthly messages. Other messages include a birthday message, a holiday message (in December), a harvest message (in November), messages acknowledging an anniversary of loss and an anniversary of hope, and 4 messages for seasonal events. The study tailored messages to reflect locally appropriate holidays, events, and celebrations, and used the local language for greetings [20]. The messages were delivered through a secure online platform, LifeWIRE (LifeWIRE Group), which was compliant with HIPAA, HiTech, and FDA regulations for electronic interactions. This platform tracked all communication, preprogrammed messages, and offered the option for individualized responses to be typed and stored for record-keeping purposes.

**Table 1:** Sample messages for caring text messages sent to intervention participants by site.

Timing	FNCH^a^ message (Albuquerque)	OHC^b^ message (Rapid City)
First day after enrollment (#1)	It was nice meeting and talking with you yesterday, [displayname]^c^. You showed great courage. Thanks for trusting me. -<staff name>^d^	Hello [displayname], It was nice meeting and talking with you yesterday. You showed great courage. Thanks for trusting me. -<staff name>
Weekly (#6)	Ya’at’eeh! Keshi! Guw’aadzi! No matter how you say “hello,” everyone is welcome at the FNCH Traditional Wellness Program. Please call (XXX) XXX-XXXX for more information. - <staff name>	Aŋpétu wašté [displayname]! Sending you positive vibes. Creator has good things for you. Smudging is a good way to stay in touch with the Creator. - <staff name>
Biweekly (#11)	Diné Prayer: “In beauty I walk, with beauty before me, behind me, above me, around me. Today I will walk out, greet the sun and the stars, everything negative will leave me, nothing will hinder me” *-*<staff name>	“One of the things my parents taught me, and I’ll always be grateful for the gift, is to not ever let anybody else define me.” -Wilma Mankiller. I believe in you, [displayname]. -<staff name>
Monthly (#16)	[displayname], being alive is fighting: “Being Indian is a combination of things. It’s your blood. It’s your spirituality. And it’s fighting for the Indian people” –Winona LaDuke -<staff name>	[displayname], I believe that the future holds many good things for you. Crazy Horse shared this vision: “I see a time of Seven Generations when all the colors of mankind will gather under the Sacred Tree of Life and the whole earth will become One Circle again.”-<staff name>
Spring (#21)	Spring is a time for prayers to help corn grow. So much will come up to greet the sun daily. Today is a new day! -<staff name>	[displayname], the Thunder Beings return to the homeland. Spring is our time to welcome their return - we can pray and give thanks for the promise of rain. -<staff name>

^a^FNCH: First Nations Community Healthsource.

^b^OHC: Oyate Health Center.

^c^[displayname] indicates that the participant’s name was used here.

^d^<staff name> indicates that the site staff signed the message with their own name.

The intervention and study design were adapted to the cultural values and context of the participating communities (eg, type of messages and topics, number of messages, and schedule) [20]. To create messaging that appealed to young urban AIs, the study teams conducted focus groups and semistructured key informant interviews with AI individuals affected by suicide, and gathered input from staff in the clinic’s behavioral health and traditional wellness departments, the project’s youth representatives, and study staff. Participants were asked to brainstorm and review text messages for their cultural appropriateness and relevance to young urban AIs in their community. The messages were then carefully reviewed and adapted by staff from the clinic’s behavioral health and traditional wellness departments, and research staff to ensure that they were consistent with the ideals of the caring texts intervention (ie, brief, supportive, and nondemanding). The messages used familiar language and were sent by research staff who had conducted the participants’ baseline assessments to establish continuity and connection.

### Data and Safety Monitoring

Site and coordinating center staff monitored participant safety. To determine and address potential suicide risk during assessments, study staff administered the University of Washington Risk Assessment Protocol to participants immediately before and after baseline and follow-up assessments [21]. If this assessment indicated moderate or severe suicidal intentions, or if the assessor has other reasons to be concerned about risk, a site-specific crisis protocol is implemented to ensure the participant receives appropriate care from a trained clinician who can more thoroughly assess their level of risk and initiate appropriate next steps.

All adverse events are identified through assessments or through care text messages, then recorded in the REDCap database, which automatically sends email alerts to the team overseeing participant safety. The site leads ensure high-risk participants receive appropriate management and follow the crisis protocol, including referral and a warm hand-off to clinic staff or a crisis line. The National Institutes of Health required a Data Safety and Monitoring Board to meet twice a year and oversee recruitment, adverse events, and protocol violations. After each meeting, the board prepares a brief report with recommendations for protocol modifications. All adverse events are reported to the universities’ Institutional Review Boards and tribal review boards, in accordance with their policies.

### Measures

A summary of all measures used in the study is available in Table 2. Unless otherwise noted, measures were administered as a survey in REDCap.

**Table 2:** Summary of outcome and measures used in the urban caring text study.

Type, topic, and domain			Scale or measure
**Primary outcomes: suicidal behaviors**			
	Suicidal ideation	Suicidal Ideation Questionnaire (SIQ-Junior)	
	Recent suicide attempts	Suicide Attempt Self-Injury Count Recent	
	Suicide-related hospitalizations	Youth Health Services Measure	
**Secondary outcomes: social connectedness**			
	Thwarted belonging, burdensomeness	Interpersonal Needs Questionnaire	
	Loneliness	Loneliness Scale from NIH^a^ Toolbox	
	Perceived rejection	Social Distress Scale	
	Objective frequency of socialization	Social Contact Questionnaire	
	Connection to community	Social Connection Venn Diagram Task	
**Potential covariates and relevant factors of interest**			
	Suicide risk	Acquired Capability for Suicide Scale and Exposure to Suicide Questionnaire	
	Stress and trauma	Breslau Post Traumatic Stress Disorder Screener, Ayers Coping Scale, and Historical Loss and Trauma Scale	
	Substance use	Addiction Severity Index, Native American Version	
	Indigenous identity	Oetting and Beauvais Orthogonal Ethnic Identification Scale	
	Overall psychological well-being	Kessler Psychological Distress Scale	
	Overall well-being	EQ-5D-5L^b^	
	Overall well-being and health care usage	Health Economics Analysis Questionnaire	

^a^NIH: National Institutes of Health.

^b^EQ-5D-5L: EuroQol 5-Dimension 5-Level.

#### Primary Outcomes

Our primary outcome of interest was suicidal behavior. While suicidal ideation is more common than suicide attempts or attempts requiring hospitalization, ideation is not necessarily a concrete event and can be more difficult to measure. For this reason, ideation, attempts, and suicide-related hospitalization were all selected to measure the same underlying risk construct. The Suicidal Ideation Questionnaire-Junior, which has been validated with AIAN individuals, measured the study’s primary outcome of suicidal ideation [22]. It consists of 15 items that evaluate thoughts of death, desire to be dead, and specific thoughts of self-harm. Scores range from 0 to 90, with scores above 32 indicating active suicidal ideation [23]. The “junior” version of the questionnaire was used to accommodate participants with lower reading levels.

The SASI count (lifetime and recent) assessed the primary outcome of suicide attempts [24]. It is a concise questionnaire that determines the first, most recent, and most severe suicide attempt or nonsuicidal self-injury. It assessed the date, method used, and intent to die (ie, clear intent to die, ambivalent intent, or no intent to die), the highest level of medical treatment received (ie, none, doctor or clinic visit, emergency room, or admission to a medical unit), and lethality of each event. The SASI also posed questions about the number of attempts for each of the 17 methods, specifying intent to die, medical treatment received, and lethality for each one. Intent to die and lethality were derived from the SASI interview [24]. A lifetime version of the instrument administered at the baseline assessment captured all historical suicide attempts or nonsuicidal self-injury. A recent version of the instrument assessed only those events that occurred in the past year, both at baseline and follow-up assessments. The adapted version of the SASI used in this study simplified the language, included methods commonly used in AIAN communities, and added an assessment of drug and alcohol use at the time of the self-injury. The SASI was administered via interview.

The primary outcome of suicide-related hospitalizations and health care encounters was measured via the Youth Health Services Measure [25]. The questionnaire collected information about the use of and satisfaction with inpatient and outpatient medical care, emergency room visits, and the use of traditional healing. It could be used to identify health care encounters related to suicidal ideation or behaviors.

#### Secondary Outcomes

We used several measures of social connectedness. The Interpersonal Needs Questionnaire describes the extent to which individuals feel connected to others (ie, belongingness) and the extent to which they perceive themselves to be a burden on other people (ie, burdensomeness) [23,26]. Previous psychometric analyses indicated that the latent variable “thwarted belongingness” significantly predicted suicidal ideation scores, supporting the measure’s construct validity [9]. The Loneliness Scale, which is part of the National Institutes of Health Toolbox Social Relationship Scales [27], assesses feelings of loneliness and exhibits both internal reliability and concurrent validity. The Social Distress Scale, also part of the National Institutes of Health Toolbox, measures perceived rejection and hostility and has good internal reliability and concurrent validity [27]. The Social Contact Questionnaire asks participants about their use of various forms of social contact, such as text messaging, email, and postal mail [28]. Finally, the Social Connection Measure asks participants to choose which of 7 Venn diagrams, each with a progressively greater proportion of overlap, best reflects their sense of closeness to their community [29]. This measure is intended to provide participants with a means of visualizing their integration within their community.

#### Covariates and Other Measures of Interest

Participants were also asked to complete surveys related to several other aspects of mental health so that they could later be assessed as possible mediators or effect measure modifiers. The Acquired Capability for Suicide Scale is a 20-item measure that quantifies one’s fearlessness about and capability to die by suicide [30-32]. It has adequate to good internal consistency (α=–.67 to .83), convergent and discriminant validity, and does not correlate with measures of depression or suicidal ideation. The Exposure to Suicide Questionnaire asks about participants’ exposure to suicide and suicide attempts made by family members, friends, or coworkers to assess possible contagion effects. This information allows a posthoc examination of the impact of exposure to suicide and suicide clusters on the intervention effects.

The Breslau Post Traumatic Stress Disorder Screening is a 7-item scale that assesses internalization of feelings following an upsetting or frightening event [33]. Questions about feelings in the past month are answered yes or no and summed to create a total score ranging from 0 to 7; higher scores indicate probable posttraumatic stress disorder. The Ayers Coping Scale is an 8-item scale that has been validated among AIAN populations to assess the frequency of using healthy coping strategies when faced with a problem [34]. The Kessler Psychological Distress Scale, also validated for administration to AIANs, is a 6-item scale that assesses the frequency of feelings of anxiety, hopelessness, depression, restlessness, and unworthiness in the past 30 days [35]. The scale is sensitive (0.36, 0.08) and specific (0.96, 0.02) with respect to predicting serious mental illness [35].

The Historical Loss and Trauma Scale is a 12-item self-report measure that assesses the frequency of thoughts regarding historical trauma (eg, “how often do you think about the loss of our traditional spiritual ways” and “how often do you think about the loss of our language”), which has high internal reliability [36-38]. The Oetting and Beauvais Orthogonal Ethnic Identification Scale poses 8 questions regarding the degree to which respondents follow the tribal and white way of life and the importance of keeping their tribal identity, values, and practices [39,40].

The EuroQol 5-Dimension 5-Level (EQ-5D-5L) is a 6-item measure that assesses respondents’ capacity to perform activities related to mobility, self-care, usual activities, and health dimensions, including pain, discomfort, anxiety, and depression, as well as overall self-reported health status [41,42]. The Health Economics Analysis Questionnaire estimates use of outpatient, inpatient, and emergency room visits; out-of-pocket costs of care; health insurance; employment and schooling benefits; income; publicly funded benefits; lifestyle behaviors (eg, substance use); and costs of participating in the study.

Self-reported drug use and drug use severity are assessed using the Addiction Severity Index, Native American Version [43]. This is a comprehensive measure of lifetime and current drug and alcohol use, as well as the impact of substance abuse on medical conditions, psychiatric well-being, employment status, and encounters with the legal system. The culturally adapted version of the measure also addresses the impact of substance use on the respondent’s ability to participate in cultural or traditional activities and ceremonies.

Demographic information included gender, sexuality, relationship status, religion, number of children, living arrangement, employment, use of the native language, and education level. Demographic features that may change are reassessed at 12 months.

The COVID-19 Impact on Well-Being Survey, a brief 5-item survey, assesses the impact of COVID-19 infection, the pandemic, and related mitigation efforts on participants’ economic, social, and overall health well-being.

We also queried intervention participants at follow-up about their experience of and satisfaction with caring texts.

#### Treatment Retention

Retention in the SBIRT program is assessed at 12 months. It is operationalized as a 3-category indicator (full, partial, and none) defining engagement for each participant in the appropriate level of care. The appropriate level of care is determined during the initial in-person session with the SBIRT behavioral therapist before enrollment in the study and is independent of study procedures. These variables will be included in sensitivity analyses if they appear unbalanced, although randomization and the large sample size should balance the severity of suicidality and intensity of appropriate intervention services across the study conditions.

### Data Analysis

#### Analysis plan

We expect that individuals receiving the culturally tailored text messages will have better outcomes than those in the treatment-as-usual control arm. Chi-square and *t* tests will be used to compare the overall percentages (binary outcomes) and mean differences (continuous outcomes) between study arms. We will conduct a sensitivity analysis using logistic or linear regression to adjust for baseline factors that are imbalanced between the intervention and control arms. Results will be presented as risk ratios and risk differences for binary outcomes, extracted from logistic regression models using marginal standardization, with 95% CIs [44,45]. Primary analyses will use an intention-to-treat approach.

#### Missing Data

Our primary analysis will rely on complete case analysis. We will conduct sensitivity analyses with multiple imputation methods. Multiple imputation through chained equations will be applied when data are missing at random. We will also conduct sensitivity analyses to exclude individuals who were randomized to receive the intervention but did not receive it per the schedule outlined in Table 2 due to technological errors or temporary lapses in access to cell phone service. These additional sensitivity analyses will improve confidence in our findings.

#### Power and Sample Size

We plan to recruit 350 people across both sites. We assume the study will have 70% retention at 12 months, yielding an analytic sample of 240 people (120 per group). With this sample, we will have 80% power to detect a 16% difference (Risk Ratio=0.47) in suicidal ideation between intervention and TAU groups, assuming that 30% of the TAU group reports suicidal ideation at follow-up. We will also be able to detect an 11% difference (Risk Ratio=0.25) in suicide attempts if 15% of the TAU group participants attempt suicide within the 12-month follow-up. There is variability in the literature regarding what constitutes a clinically meaningful effect size for reductions in suicidality and the best means of measuring such an effect (eg, degree of suicidality, reported number of attempts, etc). Regardless, we are confident in our ability to recruit a sizeable analytic sample in a difficult-to-reach population.

### Ethical Considerations

All study procedures—including consent protocol, data collection tools, and intervention materials—and subsequent modifications were reviewed and approved by the Institutional Review Boards of Washington State University (#17435), University of New Mexico (#19-401), and University of Colorado Denver (#18-0186), in addition to the Great Plains IRB (#23-R-09GP). The trial is registered on the United States National Institutes of Health Clinical Trials Registry (ClinicalTrials.gov NCT03136094). To ensure informed and voluntary participation, the study staff privately obtains consent from individuals, unless they request otherwise. The coordinator verbally explains the purpose, procedures, risks, benefits, compensation, withdrawal rights, and confidentiality of the study. While the consent form includes permission to be contacted by text message, the form does not make the nature of the intervention explicit. Potential participants are told that the clinic is testing a mobile phone program to support people who are having a difficult time, and that they will receive periodic text messages over the next 12 months. The consent process omits explanations of when and how often text messages will be sent. If participants ask for more details, they are informed that the text messages will be “periodic” and that their purpose is to check in. Keeping the participant blinded during the consent process is an important methodological feature of this study to minimize biased assessment of outcomes and maximize the validity of the results. Participants are informed that their participation is voluntary, and they can withdraw at any time without consequences. Participants at the FNCH site were compensated US $75 upon completion of the baseline visit and received an additional US $75 upon completion of the follow-up visit. Participants recruited from the OHC site were compensated US $100 upon completion of the baseline visit and received an additional US $75 upon completion of the follow-up interview.

## Results

### Overview

Participants are still being enrolled at the site in South Dakota; the site in New Mexico has concluded enrollment but is continuing with follow-up assessments. We expect to complete data collection by the Spring of 2025.

### Participant Reception

Thus far, participants have responded positively to the messages. While the messages are designed such that they do not demand anything of participants nor require a response, participants do occasionally respond. Most of these responses have been positive. Some responses are brief, with an expression of gratitude or the use of positive emojis. Other times, participants express that a particular message strongly resonated with them. Some of the messages received from participants in response to the caring texts intervention are included in Textbox 1. Names of site staff and site locations have been removed to preserve participant anonymity.

**Select participant responses to caring text messages.**

- “It was also nice talking to you, having our short conversations about yoga. It’s always great to spill information out to feel less worried than before. Thank you for working with <site>. :)”
- “Wow, love that quote makes so much sense, thanks for sharing.”
- “Cool. I like that one.”
- “Thank you for listening.”
- “I hope all is well with you. Thank you for the great positive feedback throughout each day. Your time is much appreciated!”
- “Cool. Going through that right now. Thanks for the quote.”
- “Thank you so much for putting in the time to relay the resources to me. I appreciate it so much, and thank you for sharing your light.”
- “Thank you, <staff name>! Thank you for listening.”
- “Hi! That’s a great outlook. I’ll use that today. Hope you have a good week too!”
- “Today is a new day indeed, hope you are having a good day!”
- “Happy New Year, <staff name>, thank you for all the positive words. I do appreciate it.”
- “Good morning, thanks for the words of encouragement and wisdom.”
- “I hope you have a great one as well. Thanks for keeping in contact.”
- “Thank you, it made me very proud to give my answers, knowing people can know more about the struggles our people face. I hope I can help more one day.”
- “Nothing that I can think of at the moment has been doing well, but it’s good to know I have support and people to reach out to if need be. Thanks, you guys! Have a great rest of your day, <staff name>”
- “Thank you. It’s hard to talk about that stuff. But I felt I could trust you.”
- “I love that saying, and it helped put a smile on my face for the day.”

## Discussion

### Principal Findings

This study represents an opportunity to prevent or reduce suicidality among AI young adults in urban areas across the country [9-11,15,28]. We anticipate finding that those individuals who received the caring text messages will show reduced indications of suicidality (primary outcome) and increased feelings of social connectedness (secondary outcomes) compared to those in the TAU cohort. This is because the caring texts intervention has strong theoretical foundations [9,10] and is supported by evidence-based practice guidelines [11,15,28].

In addition to being theoretically grounded and supported by other evidence-based practices, we have endeavored to develop an intervention that could be implemented at scale with limited need for involvement on the part of behavioral health specialists on a day-to-day basis. The intervention is structured and delivered in such a way that it can be integrated into primary care, emergency departments, or other services not explicitly designed to manage mental health care. There is mounting support for integrating behavioral health services into primary care, which can reduce stigma and improve access to limited behavioral health resources [46-51]. This is particularly pertinent for young adults who may be trying to access care from an already overburdened IHS.

### Dissemination Plan

We believe that disseminating findings back to the community is a crucial component of community-based participatory research. To this end, we have planned several opportunities for dissemination within the scientific community and among urban AI communities more broadly. The findings from the study will be published in an academic journal following the conclusion of data collection and analysis. In addition, we have participated and will continue to participate in health fairs hosted near both study sites. Previous attendance has included sharing information regarding this trial and other behavioral health resources. Future presentations will include sharing the results of the completed trial back to the community. In addition to these health fairs, the staff at the South Dakota site routinely participates in local radio shows to share information on various health topics and ongoing research projects. They plan to dedicate one segment to discussing the findings of the urban caring text messages study.

### Conclusions

The intervention was culturally adapted by members of each participating urban native community. The inclusion of different constituents, such as patients at risk of suicide and behavioral health providers, in the cocreation of the intervention ensures the relevance and appropriateness of the intervention’s content, including the text messages. The text messages are designed to be culturally appropriate and increase social support and connectedness without imposing demands on the recipient. By studying whether caring texts help to reduce suicidal ideation and behaviors, and improve social connectedness as well as connection to Indigenous identity, this trial will provide evidence to inform whether primary care clinics serving AIAN young adult should integrate caring texts into their services.

## Appendix

Multimedia Appendix 1 Full schedule for caring text messages sent to intervention participants by site.

Multimedia Appendix 2 SPIRIT checklist.

## Abbreviations

AI: American Indian
AIAN: American Indian and Alaska Native
EHR: electronic health record
EQ-5D-5L: EuroQol 5-Dimension 5-Level
FNCH: First Nations Community HealthSource
HLQ: Healthy Lifestyle Questionnaire
IHS: Indian Health Service
OHC: Oyate Health Center
SASI: Suicide Attempt Self-Injury
SBIRT: screening, brief intervention, and referral to treatment
TAU: Treatment as Usual
