# Supplementary material for: Caring Text Messages for Suicide Prevention in Urban American Indian Youth: Protocol for a Randomized Controlled Trial
Source: JMIR Res Protoc. 2025 Sep 26;14:e71344. doi: 10.2196/71344 (PMC12514422; doi:10.2196/71344)
Supplement: Multimedia Appendix 1 [file resprot_v14i1e71344_app1.docx]

| **Appendix Table.** Full Schedule for Caring Text Messages Sent to Intervention Participants by Site | | |
| --- | --- | --- |
| **Timing** | **FNCH/Albuquerque Message** | **OHC/Rapid City Message** |
| 1^st^ day after enrollment (1) | It was nice meeting and talking with you yesterday, [displayname]. You showed great courage. Thanks for trusting me. – <staff name> | Hello [displayname], It was nice meeting and talking with you yesterday. You showed great courage. Thanks for trusting me. –<staff name> |
| 2^nd^ day after enrollment (2) | Hey [displayname], continue to live the story no one else can live - your story. Thanks for entrusting me with your story. - <staff name> | Hey there [displayname], just a quick reminder that I’m available M-F 10AM to 3PM, but your resource card lists more places to get help overnight or during the weekend. – <staff name> |
| 3^rd^ day after enrollment (3) | Hey there [displayname], just a quick reminder that I’m available M-F 8AM to 5PM but check your resource card for more places to get help overnight or during the weekend. Thanks for understanding. - <staff name> | Híŋhaŋni wašté [displayname]! “When you are lonely, I wish you love. When you are down, I wish you joy. When you are troubled, I wish you peace. When things are complicated, I wish you simple beauty. When things look empty, I wish you hope.” by Mercheeko Macrone  –<staff name> |
| Weekly (4) | Hello, here is an inspirational quote for today from a Native Elder: “Good health is the ability to transform pain into wisdom” – Dr Larry Emerson (Diné) Wishing you good health today. - <staff name> | Híŋhaŋni wašté [displayname], I hope this finds you in a good place. “The old people came literally to love the soil and they sat or reclined on the ground with a feeling of being close to a mothering power. It was good for the skin to touch the earth and the old people liked to remove their moccasins and walk with bare feet on the sacred earth. . . . The soil was soothing, strengthening, cleansing, and healing. . . . Wherever the Lakota went, he was with Mother Earth. No matter where he roamed by day or slept by night he was safe with her.” - Luther Standing Bear, Land of the Spotted Eagle – <staff name> |
| Weekly (5) | Hi [displayname], wishing you a peaceful day - <staff name> | Aŋpétu wašté [displayname]! Confucius says, “It does not matter Aŋpétu wašté [displayname]! Confucius says, “It does not matter how slow you go as long as you do not stop.” Thank you for coming this far. – <staff name> |
| Weekly (6) | Ya’at’eeh! Keshi! Guw’aadzi! No matter how you say “hello” everyone is welcome at the FNCH Traditional Wellness Program. Please call (XXX) XXX-XXXX for more information. - <staff name> | Aŋpétu wašté [displayname]! Sending you positive vibes. Creator has good things for you. Smudging is a good way to stay in touch with Creator. – <staff name> |
| Weekly (7) | An arrow can only be released by first pulling it back. When life pulls you back, it simply means a chance to launch into something amazing. *-* <staff name> | Híŋhaŋni wašté [displayname], I hope this finds you in a good place. I am thinking of you and would like to share: “Healing does not mean the damage never existed. It means the damage no longer controls us.” by Shah Rukh Khan –<staff name> |
| Weekly (8) | “What’s your clan?” Our ancestors created clans (and villages, lodges, societies) to remind us that, as Native people, we are surrounded by family. You are our family & you belong. *-* <staff name> | Aŋpétu wašté [displayname]! “We delight in the beauty of the butterfly, but rarely admit the changes the butterfly had to go through to achieve that beauty.” - Maya Angelou.  We too go through many changes. –<staff name> |
| Weekly (9) | "I found a family, at ceremonies, in the language." FNCH’s traditional wellness & healing helps people find their ceremonial family. Here is the link if you are interested. - <staff name> | Híŋhaŋni wašté [displayname]. I try to look for something positive each day, even though some days I must look a little harder. – <staff name> |
| Biweekly (10) | It’s okay to be human and feel your feelings. Hope you are well today. | Aŋpétu wašté [displayname]! I thought of you today. Humor is one thing in our culture that we share to help us get through the day. I am here for you. – <staff name> |
| Biweekly (11) | Diné Prayer: "In beauty I walk, with beauty before me, behind me, above me, around me. Today I will walk out, greet the sun and the stars, everything negative will leave me, nothing will hinder me" *-*<staff name> | "One of the things my parents taught me, and I'll always be grateful for the gift, is to not ever let anybody else define me." - Wilma Mankiller  I believe in you, [displayname]. – <staff name> |
| Biweekly (12) | Hey there [displayname], when I’m in a funk, I ask myself these questions: “What does my body need? What does my mind need? What does my spirit need?” Hope you are well today. - <staff name> | Hey there [displayname], when I’m in a funk, I ask myself these questions: “What does my body need? What does my mind need? What does my spirit need?” – <staff name> |
| Biweekly (13) | There is beauty in the fields, the clouds, the brown earth, the weather, the rivers and nature. And we are all a part of it. - <staff name> | Aŋpétu wašté [displayname]. I would like to share a Lakota prayer with you… “Teach to trust so that I may enter my sacred space & love beyond my fear & to walk in balance with the passing of each glorious sun.”  – <staff name> |
| Biweekly (13) | This journey called life, with all its ups & downs, twists & turns, is to be embraced and respected. Hope your day is going well. *-* <staff name> | Hello [displayname]! Take comfort today, I am thinking and caring about you and keeping you in my thoughts. – <staff name> |
| Biweekly (14) | Talking to someone supportive helps if we’re feeling down or going through hard times. There are phone numbers listed on the resource card to help in this way. *-* <staff name> | “Let us meet each other with a smile, for the smile is the beginning of love.” -Mother Theresa. Hope your day is going well. – <staff name> |
| Monthly (15) | Remember all the incredible things you've **survived** and know healing is possible. *-* <staff name> | Aŋpétu wašté [displayname]! Patience is the calm acceptance that things happen in a different order than the one we have in mind. Sharing some positive thoughts for encouragement. You are important. – <staff name> |
| Monthly (16) | [displayname], being alive is fighting: "Being Indian is a combination of things. It's your blood. It's your spirituality. And it's fighting for the Indian people" ~ Winona LaDuke *-* <staff name> | [displayname], I believe that the future holds many good things for you. Crazy Horse shared this vision: "I see a time of Seven Generations when all the colors of mankind will gather under the Sacred Tree of Life and the whole earth will become One Circle again." – <staff name> |
| Monthly (17) | A bad day doesn’t mean a bad life. Sometimes, life just blesses us with a different point of view. I find it helpful to talk with someone supportive during this time. Your care card lists a variety of resources. *-* <staff name> | Híŋhaŋni wašté [displayname]! Sometimes, finding someone just to listen is helpful. The Oyate Health Center caring professionals can do just that. They can be reached at (###) ###-####, and someone from their team will gladly help you. – <staff name> |
| Monthly (18) | Checking in to say, hi. I’ve learned to take a few minutes every day to give myself some credit for good in the day. Writing it out helps me maintain a positive mood. Hope you have a good week. - <staff name> | Hello [displayname], I wanted to let you know that you are not alone. There are many people who care about you. As always, I am here for you. Have a great day. –<staff name> |
| Monthly (19) | Words I say to assure myself: “I am good enough. I matter. I accept what the day brings.” I hope you are well today. - <staff name> | Aŋpétu wašté [displayname]. Just to let you know I am thinking of you. I wish you a day with a lot of positivity. – <staff name> |
| Monthly (20) | Pray, smudge, meditate- set the tone for the day. It’s a good day to be indigenous *-* <staff name> | Congratulations, [displayname]! Creator has blessed you with another day. Smudging can enhance the mystery of creator and change the negative vibes into positive vibes. – <staff name> |
| Spring (21) | Spring is a time for prayers to help corn grow. So much will come up to greet the sun daily. Today is a new day! *-* <staff name> | [displayname], the Thunder Beings return to the homeland. Spring is our time to welcome their return – we can pray and give thanks for the promise of rain. – <staff name> |
| Summer (22) | “Without the rain, there would be no rainbow." May the summer season bring you many rainbows as they are a sign of hope. *-* <staff name> | “And as he spoke of understanding, I looked up and saw the rainbow leap with flames of many colors over me” - quote by Black Elk. May the summer season bring you many rainbows as they are a sign of hope. – <staff name> |
| Fall (23) | There is beauty in the changing seasons: “Coyote looked at the mountains and said, ‘We'll see you again.’ And prayed for safety, strength, and the ability to see beauty.” -Simon Ortiz *-* <staff name> | There is beauty in the changing seasons. “Coyote looked at the mountains and said, ‘We'll see you again.’ And prayed for safety, strength, and the ability to see beauty.” – by Simon Ortiz  – <staff name> |
| Winter (24) | “How many winters have you survived?” Winters remind us of where we’ve been and have yet to discover. We can use this season to rest, heal, learn and prepare for the spring. *-* <staff name> | Aŋpétu wašté [displayname]. Winters remind us of where we’ve been and have yet to discover. We can use this season to rest, heal, learn, and prepare for the spring. – <staff name> |
| Birthday (25) | Your age is a testimonial of how much life you have enjoyed & how much is still left to explore. Happy Birthday Friend! - Name | Nitȟá-aƞpétu wašté [displayname]! Wishing you a very Happy Birthday and a year filled with love, adventure, and prosperity. Happy Birthday Friend! – <staff name> |
| November (26) | Sharing a quote for Native American Heritage Month: “I am strong for my ancestors, I am strong for my people, I am strong for the ones to come, I am here because of my ancestor’s resilience” -<staff name> | Hello [displayname]! Harvest season is here. In Fall we can enjoy eating fresh vegetables and wild fruit. Sending wishes for a good day for you. – <staff name> |
| Anniversary of loss (27) | "I'll always feel you close to me and though you're far from sight, I'll search for you among the stars that shine at night."  Thinking about you and your loss. Sending courage and hope your way. *-* <staff name> | "I'll always feel you close to me and though you're far from sight, I'll search for you among the stars that shine at night" – Anonymous author Thinking about you and your loss [displayname]. Sending courage and hope your way. – <staff name> |
| December Holiday (28) | Whether you call, text, or meet up in-person, we all need a shoulder to lean on & someone to listen to us. Reaching out to supportive friends or family can be an option. Happy holidays, <staff name>*.* | [displayname], Whether we call, text, or meet up in-person, many of us need a shoulder to lean on or someone to listen. May love and light fill your home and heart. Happy holidays, <staff name> |
| Anniversary of hope (29) | [displayname], I am happy for you today. May you keep moving forward in your journey, <staff name>*.* | [displayname], I am happy for you today. “Hope is being able to see that there is light despite all the darkness.” by Desmund Tutu  – <staff name> |
| FNCH–First Nations Community Healthsource. OHC—Oyate Health Center.  [displayname] indicates that the participant's name was used here.  <staff name> indicates that site staff signed the message with their own name. | | |
